# Meta-analysis of the efficacy of Da Vinci robotic or laparoscopic distal subtotal gastrectomy in patients with gastric cancer

**DOI:** 10.1097/MD.0000000000027012

**Published:** 2021-08-27

**Authors:** Zibo Zhang, Xiaolin Zhang, Yu Liu, Yong Li, Qun Zhao, Liqiao Fan, Zhidong Zhang, Dong Wang, Xuefeng Zhao, Bibo Tan

**Affiliations:** a Hebei Medical University Fourth Affiliated Hospital and Hebei Provincial Tumor Hospital, Third Department of Surgery, Shijiazhuang, Hebe, China; b Hebei Medical University, School of Public Health, Shijiazhuang, Hebei, China.

**Keywords:** distal gastrectomy, gastric cancer, laparoscopy, meta-analysis, robotic

## Abstract

**Background:**

Robotic-assisted gastrectomy has been used for treating gastric cancer since 2002. This meta-analysis was conducted to systematically evaluate the efficacy of Da Vinci robotic distal subtotal gastrectomy (RDG) or laparoscopic distal subtotal gastrectomy (LDG) in patients with gastric cancer.

**Methods:**

We conducted searches in domestic and foreign databases, and collected literature in Chinese and English on the efficacy of RDG and LDG for gastric cancer that have been published since the inception of the database. RevMan 5.4.1 was used for meta-analysis and drawing and Stata14.0 was used for publication bias analysis.

**Results:**

A total of 3293 patients in 15 studies were included, including 1193 patients in the RDG group and 2100 patients in the LDG groups respectively. The meta-analysis showed that intraoperative blood loss was significantly lower and the number of resected lymph nodes was higher in the RDG group compared to that in the LDG group. In addition, the times to first postoperative food intake and postoperative hospital stay were shortened, and there was a longer length of distal resection margin and prolonged duration of operation. No significant differences were found between the 2 groups with respect to the first postoperative anal exhaust time, length of proximal resection margin, total postoperative complication rate, postoperative anastomotic leakage rate, incidence of postoperative gastric emptying disorder, pancreatic fistula rate, recurrence rate, and mortality rate.

**Conclusion:**

RDG is a safe and feasible treatment option for gastric cancer, and it is non-inferior or even superior to LDG with respect to therapeutic efficacy and radical treatment.

## 1 Introduction

Gastric cancer, a malignant tumor of the digestive tract, is the fourth most common cancer worldwide and is more common in developing countries.^[1]^ Surgery and chemotherapy are the mainstay of treatment for gastric cancer, and surgical resection is the predominant approach. The first laparoscopic-assisted gastrectomy (LG) was reported by Kitano et al^[2]^ in 1994. With the development of minimally invasive surgical approach for gastric cancer, laparoscopic instruments exhibited several disadvantages, such as limited motion in a confined space, two-dimensional view, and obscure vision during surgery. Therefore, based on laparoscopic technique, Intuitive Surgical has developed the Da Vinci robotic surgical system, which was first applied for gastrectomy in 2002 by Hashizume et al.^[3]^ Subsequently, robotic-assisted radical gastrectomy (RG) has been widely performed in various countries, and a large number of clinical studies have verified its safety and prognostic effects. Currently, there have been multiple meta-analyses on the comparison of short-term effects of LG and RG for gastric cancer, and, most of the articles included patients undergoing total gastrectomy. However, robotic distal subtotal gastrectomy (RDG) mainly refers to a surgical method of distal gastrectomy using a Da Vinci robotic device. Despite several articles that included patients undergoing distal gastrectomy, some disparities are present in some results and conclusions. In addition, with the continuous development of Da Vinci robotic surgical system, research data on RG are constantly being updated. Therefore, in this study, we performed a meta-analysis of available comparative studies to compare the efficacy of laparoscopic distal subtotal gastrectomy (LDG) and RDG for gastric cancer.

## 2 Materials and methods

Ethical approval and patient consent were not required because this was a meta-analysis of previously published studies. This meta-analysis is conducted and reported in adherence to PRISMA (Preferred Reporting Items for Systematic Reviews and Meta-Analyses).

### 2.1 Search strategies

We systematically searched the databases of PubMed, Embase, Web of Science, the Cochrane library, full-text database of digital journals (Wanfang data), full-text database of Chinese journals (CNKI), and other databases for the comparison of the efficacy of LDG and RDG for gastric cancer. For each database, we used the following 5 keywords: “robotic”, “laparoscopy”, “gastrectomy”, “gastric cancer”, “gastric neoplasms” in English as well as “robots”, “laparoscopy”, and “gastric cancer” in Chinese from the inception of each database. In addition, we expanded the scope of retrieval databases through the “extension in Chinese and English” function to avoid missed retrieval. Finally, in PubMed, 336 documents were retrieved using the keywords “robotic,” “gastrectomy,” and ”laparoscopy;” 315 documents were retrieved using the keywords “robotic,” “gastric cancer,” and ”laparoscopy;” 282 documents were retrieved using the keywords “robotic,” “gastric neoplasms,” and ”laparoscopy.” In Embase, 452 documents were retrieved using the keywords “robotic,” “gastrectomy,” and ”laparoscopy;” 379 documents were retrieved using the keywords “robotic,” “gastric cancer,” and ”laparoscopy;” and 363 documents were retrieved using the keywords “robotic,” “gastric neoplasms,” and ”laparoscopy.“ In Web of science, 91 documents were retrieved using the keywords ”robotic,“ ”gastrectomy,” and “laparoscopy;” 82 documents were retrieved using the keywords ”robotic,“ ”gastric cancer,” and “laparoscopy;” and 17 documents were retrieved using the keywords ”robotic,“ ”gastric neoplasms,” and “laparoscopy”.

### 2.2 Inclusion and exclusion criteria

Studies were included if they fulfilled the following criteria: randomized or non-randomized controlled studies published on RDG and LDG for the treatment of gastric cancer; studies focusing only on patients with gastric cancer undergoing distal radical gastrectomy; at least 1 item of data provided on clinical efficacy comparison of RDG and LDG; studies with original data; for continuous variables, mean and standard deviation or mean and extreme values provided; for count data, the number of incidents and total number of samples provided; for binary variables, a combined odds ratio (OR) value and 95% confidence interval (CI) provided or a regression coefficient that could be converted into an OR value and 95% CI and its standard deviation provided. The following studies were excluded: comparative study of non-LDG and RDG; studies on patients only undergoing palliative subtotal gastrectomy, cytoreductive surgery, gastric volume reduction surgery, or short circuit surgery; studies without necessary comparison data; published literature with duplicated data.

### 2.3 Data extraction

The data were extracted independently by 2 researchers. Any disagreement was resolved through discussion or judged by the third researcher. The extracted data included the following: general information, including author, year of literature publication, research type, sample size, body mass index; outcome indicators, including duration of operation and intraoperative blood loss, the number of resected lymph nodes, length of proximal and distal resection margins, postoperative hospital stay, first postoperative anal exhaust time and time to first postoperative food intake, postoperative total complication rate, postoperative anastomotic leakage rate, postoperative gastrointestinal emptying disorder rate, postoperative pancreatic fistula rate, recurrence rate, and mortality rate.

### 2.4 Literature quality evaluation

In this study, the modified Newcastle Ottawa Scale recommended by Ji et al^[4]^ was used to evaluate the quality of the selected studies. Quality evaluation was performed in 3 aspects, including the research design (I: presence or absence of randomized controlled study; II: presence or absence of inclusion criteria; III: presence or absence of a total sample size of over 100 cases), comparability (a: sex; b: age; c: body mass index; d: tumor location; e: tumor staging), and result evaluation (lymph node dissection). Each item that met the criteria was marked “∗”, with a total of 9 “∗”; the score of 6 “∗” points and above suggested high-quality of the included studies.

### 2.5 Statistical analysis

RevMan 5.4.1 (The Cochrane Collaboration, Oxford, England) was used for meta-analysis and drawing. The Software Stata14.0 (StataCorp LP, Texas, United States) was used for publication bias analysis. Count data uses OR value, continuous variables were evaluated to obtain weighted mean difference (WMD) for comparison and meta-analysis, and the effect size was calculated with 95%CIs for each outcome. Statistical heterogeneity among studies was evaluated by using the *I*^2^ and the Q tests. If the *I*^2^ value was >50% and *P* < .1, it indicated heterogeneity, and a random-effects model was used for meta-analysis. We used a fixed-effects model if no heterogeneity was found between studies when *I*^2^ < 50% and *P* > .1. The original data, which were represented by the median and interquartile range, were converted to the mean and standard deviation. Sensitivity analysis was performed with a one-by-one exclusion method.

## 3 Results

**Figure 1:**
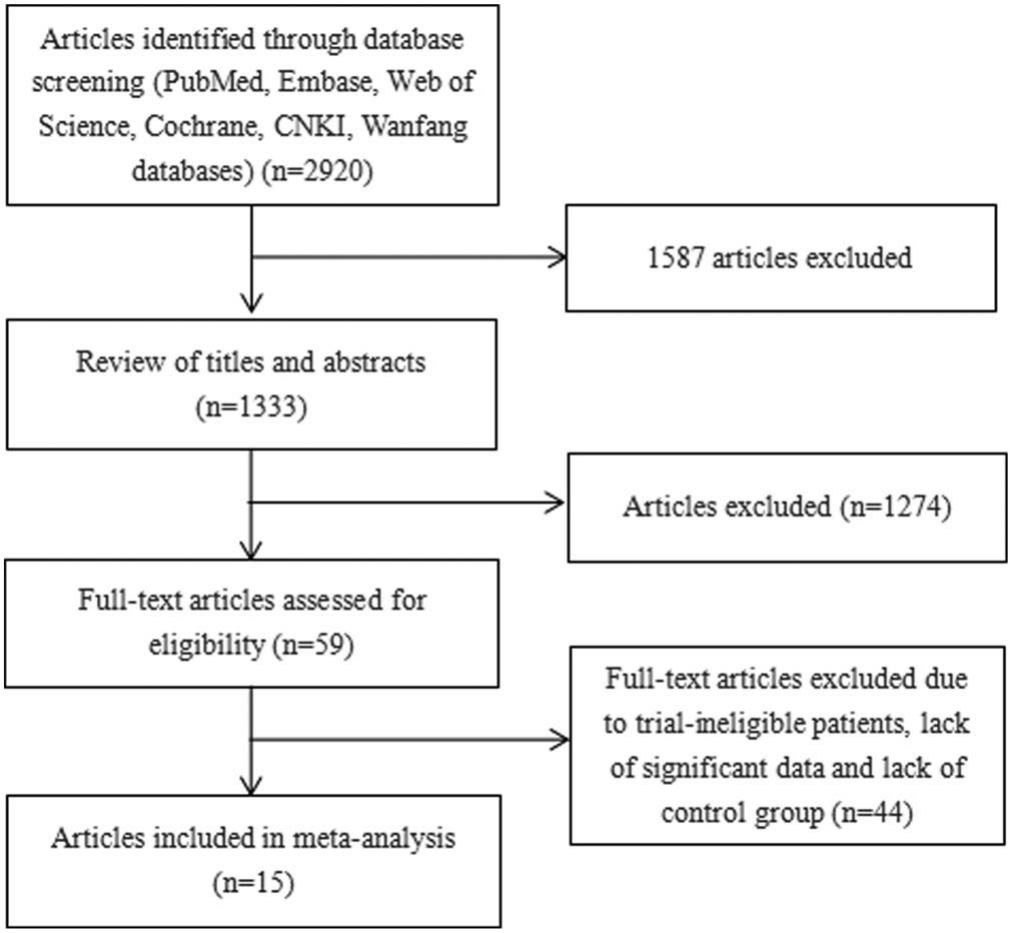
Flow diagram of the study selection process.

The initial search of databases identified 2920 studies, and 1587 duplicate articles were excluded. Another 1274 studies were excluded after a combined review of the titles and abstracts. After careful review of full-text articles, 44 studies were excluded due to ineligible patients, lack of significant data, and lack of control group. Finally, we obtained 15 articles, consisting of 8 studies in Chinese and 7 studies in English, with a total of 3293 patients for inclusion. Approximately 1193 patients were in the RDG group and 2100 patients in the LDG group (Fig. 1). The basic characteristics of the selected literature are shown in Tables 1 and 2. The literature quality evaluation is shown in Table 3.

**Table 1:** General characteristics of included articles.

Reference (year)	Country	Study period	Design	No. of patients (n)		Population	LND
				RDG	LDG		
Eom et al (2012)	Korea	2009–2010	NRCT	30	60	EGC+AGC	D1+D2
Park et al (2012)	Korea	2010–2011	NRCT	30	120	EGC+AGC	D1+
Zhao et al (2013)	China	2012	NRCT	30	30	EGC+AGC	D1+D2
Kim et al (2013)	Korea	2003–2013	NRCT	172	481	EGC+AGC	D1+D2
Xue et al (2014)	China	2012–2014	NRCT	50	60	EGC+AGC	D1+D2
Kim et al (2015)	Korea	2003–2013	NRCT	87	288	EGC+AGC	D1+D2
Lee et al (2015)	Korea	2003–2010	NRCT	133	267	EGC+AGC	D2
Xue et al (2016)	China	2012–2014	NRCT	35	35	AGC	D2
Cianchi et al (2016)	Italy	2008–2015	NRCT	30	41	EGC+AGC	D1+D2
Teng et al (2017)	China	2016–2017	NRCT	41	58	EGC+AGC	D1+D2
Li et al (2018)	China	2015–2017	NRCT	50	56	AGC	D2
Liu et al (2018)	China	2012–2017	NRCT	156	111	EGC+AGC	D1+D2
Peng et al (2018)	China	2015–2017	NRCT	120	120	EGC+AGC	D2
Peng et al (2018)	China	2015–2017	NRCT	60	60	EGC+AGC	D2
He et al (2019)	China	2016–2018	NRCT	146	127	AGC	D2
Matsunaga et al (2020)	Japan	2011–2017	NRCT	23	186	EGC+AGC	D1+D2

AGC = advanced gastric cancer, EGC = early gastric cancer, LDG = laparoscopic-assisted distal subtotal gastrectomy, LND = lymph node dissection, NRCT = non-randomized controlled trials, RDG = robotic-assisted distal subtotal gastrectomy.

**Table 2:** General characteristics of included articles (to be continued).

Reference (year)	Sex (M/F)		Age (years) (mean–SD)		BMI (kg/m^2^)		Resection extent
	RDG	LDG	RDG	LDG	RDG	LDG	
Eom et al (2012)	21/9	41/21	52.8 (28–74)^∗^	57.9 (34–78)^∗^	24.2 (17–35)^∗^	24.1 (19–30)^∗^	Distal subtotal
Park et al (2012)	18/12	65/55	50 (45–54)^∗^	55 (45–64)^∗^	23.9 (21.5–25.2)^∗^	23.6 (21.9–25.5)^∗^	Distal subtotal
Zhao et al (2013)	22/8	23/7	71.8 ± 5.7	72.4 ± 5.2	23.6 ± 1.6	23.9 ± 1.8	Distal subtotal
Kim et al (2013)	103/69	294/187	55.2 ± 13.0	61.3 ± 11.9	23.7 ± 2.9	23.6 ± 2.9	Distal subtotal
Xue et al (2014)	37/13	42/22	56.9 ± 10.6	56 ± 13.8	24.4 ± 2.8	23.8 ± 3.7	Distal subtotal
Kim et al (2015)	46/41	170/118	54.1 ± 12	60.5 ± 11	24.1 ± 3.4	24 ± 3.4	Distal subtotal
Lee et al (2015)	85/48	154/113	56.3 ± 13.2	59.2 ± 11.7	23.2 ± 2.7	23.7 ± 2.8	Distal subtotal
Xue et al (2016)	26/9	25/10	59.2 ± 9.6	56.2 ± 14.1	24.6 ± 2.9	23.4 ± 2.3	Distal subtotal
Cianchi et al (2016)	14/16	19/22	73 (45–86)^∗^	74 (40–87)^∗^	27 (23–38)^∗^	26 (23–30)^∗^	Distal subtotal
Teng et al (2017)	29/12	40/18	58 ± 11.2	59 ± 9.8	24.25 ± 2.01	24.64 ± 2.8	Distal subtotal
Li et al (2018)	35/15	37/19	65.6 ± 8.3	66 ± 7.4	24.3 ± 2.1	24.6 ± 2.6	Distal subtotal
Liu et al (2018)	114/42	76/35	59.2 ± 11.5	60.2 ± 11.6	24.1 ± 3.1	23.4 ± 3.3	Distal subtotal
Peng et al (2018)	78/42	82/38	–	–	24.37 ± 3.0	24.45 ± 3.41	Distal subtotal
Peng et al (2018)	39/21	42/18	–	–	24.47 ± 2.74	24.21 ± 3.57	Distal subtotal
He et al (2019)	103/43	86/41	60.2 ± 10.5	59.3 ± 11.6	21.9 ± 3.2	22.1 ± 3.1	Distal subtotal
Matsunaga et al (2020)	15/8	127/59	66.6 ± 11	68.9 ± 11.4	22.7 ± 1.9	22.5 ± 3.0	Distal subtotal

BMI = body mass index, F = female, LDG = laparoscopic-assisted distal subtotal gastrectomy, M = male, RDG = robotic-assisted distal subtotal gastrectomy, SD = standard deviation.

∗ Median (range).

**Table 3:** Quality scores of included articles.

Reference (year)	Research design			Intra-group comparison					Evaluation of results (the number of resected lymph nodes)	Score
	I	II	III	a	b	c	d	e		
Eom et al (2012)	–	^∗^	–	^∗^	^∗^	^∗^	^∗^	^∗^	^∗^	7^∗^
Park et al (2012)	–	^∗^	^∗^	^∗^	^∗^	^∗^	^∗^	^∗^	^∗^	8^∗^
Zhao et al (2013)	–	^∗^	–	^∗^	^∗^	^∗^	^∗^	^∗^	^∗^	7^∗^
Kim et al (2013)	–	^∗^	^∗^	^∗^	^∗^	^∗^	–	^∗^	^∗^	7^∗^
Xue et al (2014)	–	^∗^	^∗^	^∗^	^∗^	^∗^	–	^∗^	^∗^	7^∗^
Kim et al (2015)	–	^∗^	^∗^	^∗^	^∗^	^∗^	–	^∗^	^∗^	7^∗^
Lee et al (2015)	–	^∗^	^∗^	^∗^	^∗^	^∗^	–	^∗^	^∗^	7^∗^
Xue et al (2016)	–	^∗^	–	^∗^	^∗^	^∗^	–	^∗^	^∗^	6^∗^
Cianchi et al (2016)	–	^∗^	–	^∗^	^∗^	^∗^	^∗^	^∗^	^∗^	7^∗^
Teng et al (2017)	–	^∗^	–	^∗^	^∗^	^∗^	–	^∗^	^∗^	6^∗^
Li et al (2018)	–	^∗^	^∗^	^∗^	^∗^	^∗^	^∗^	–	^∗^	7^∗^
Liu et al (2018)	–	^∗^	^∗^	^∗^	^∗^	^∗^	–	^∗^	^∗^	7^∗^
Peng et al (2018)	–	^∗^	^∗^	^∗^	–	^∗^	–	^∗^	^∗^	6^∗^
Peng et al (2018)	–	^∗^	^∗^	^∗^	–	^∗^	–	^∗^	^∗^	6^∗^
He et al (2019)	–	^∗^	^∗^	^∗^	^∗^	^∗^	–	^∗^	^∗^	7^∗^
Matsunaga et al (2020)	–	^∗^	^∗^	^∗^	^∗^	^∗^	–	^∗^	–	6^∗^

### 3.1 Meta-analysis results

#### 3.1.1 Duration of operation

**Figure 2:**
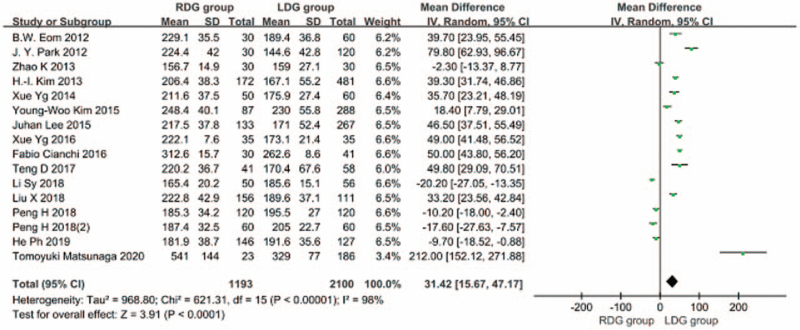
Comparison of duration of operation.

Fifteen articles^[5–19]^ reported on duration of operation. The heterogeneity between studies was significant (*I*^2^ = 98%; *P* < .1), and a random-effects model was used. The results showed that the duration of surgery was longer in the RDG group than in the LDG group [WMD, 31.420 (95%CI, 15.674–47.167); *P* < .05] (Fig. 2).

#### 3.1.2 Intraoperative blood loss

**Figure 3:**
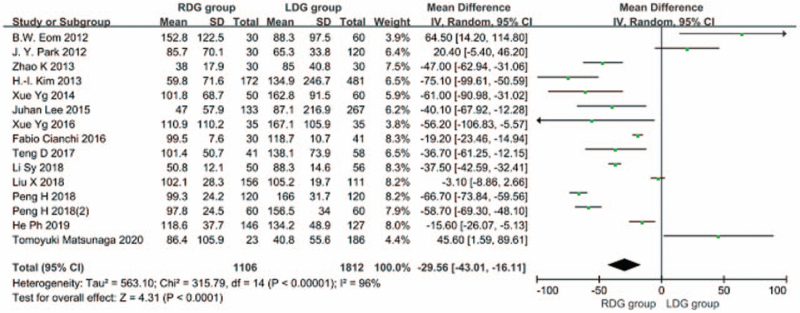
Comparison of intraoperative blood loss.

Fourteen articles^[5–9,11–19]^ reported on intraoperative blood loss. The heterogeneity between studies was significant (*I*^2^ = 96%; *P* < .1), and a random-effects model was used. The results showed that intraoperative blood loss was significantly lower in the RDG group than in the LDG group [WMD, –29.561 (95%CI, –43.010 to –16.111); *P* < .05] (Fig. 3).

#### 3.1.3 Number of resected lymph nodes

**Figure 4:**
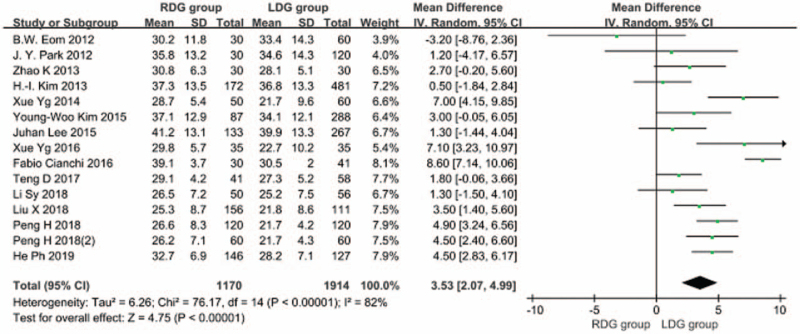
Comparison of the number of resected lymph nodes.

Fourteen articles^[5–18]^ reported on the number of resected lymph nodes. The heterogeneity between studies was significant (*I*^2^ = 82%; *P* < .1), therefore, a random-effects model was used. The results showed that the number of resected lymph nodes was higher in the RDG group than in the LDG group [WMD, 3.528 (95%CI, 2.071–4.985); *P* < .05] (Fig. 4).

#### 3.1.4 The time to first postoperative food intake

**Figure 5:**
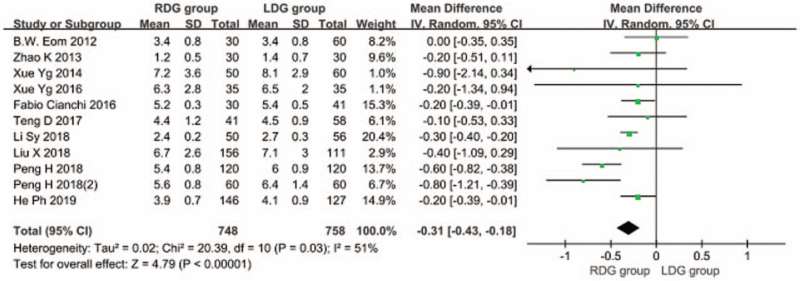
Comparison of time to first postoperative food intake.

Ten articles^[5,7,9,12–18]^ reported the time to first postoperative food intake. There was heterogeneity between studies (*I*^2^ = 51.0%; *P* < .1), and a random-effects model was used. The results showed that the time to first postoperative food intake was shorter in the RDG group than in the LDG group [WMD, –0.306 (95%CI, –0.430 to –0.181); *P* < .05] (Fig. 5).

#### 3.1.5 Length of distal resection margin

**Figure 6:**
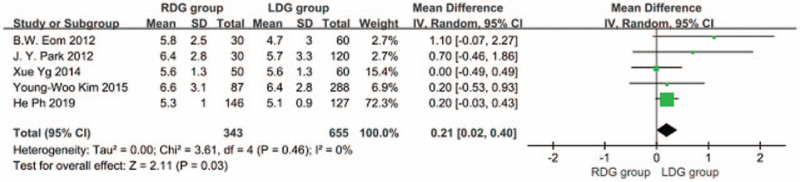
Comparison of the length of distal resection margin.

Five articles^[5,6,9,10,18]^ reported on the length of the distal resection margin. The heterogeneity between studies was significant (*I*^2^ = 0.0%; *P* > .1), therefore, a fixed-effects model was used. The result showed that the length of distal resection margin was greater in the RDG group than in the LDG group. [WMD, 0.207 (95%CI, 0.015–0.398); *P* = .03] (Fig. 6).

#### 3.1.6 Postoperative hospital stay

**Figure 7:**
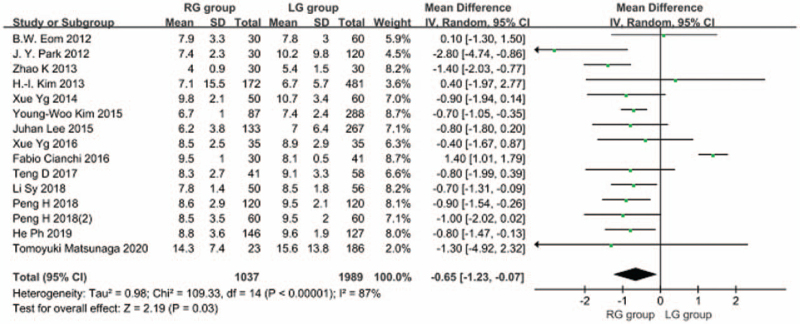
Comparison of the length of postoperative hospital stay.

Fourteen articles^[5–15,17–19]^ reported length of hospital stay, and there was heterogeneity between studies (*I*^2^ = 87%; *P* < .1); therefore, a random-effects model was used. The results showed that the length of hospital stay was shorter in the RDG group than in the LDG group [WMD, –0.649 (95%CI, –1.232 to –0.067); *P* = .03] (Fig. 7).

#### 3.1.7 The first postoperative anal exhaust time

**Figure 8:**
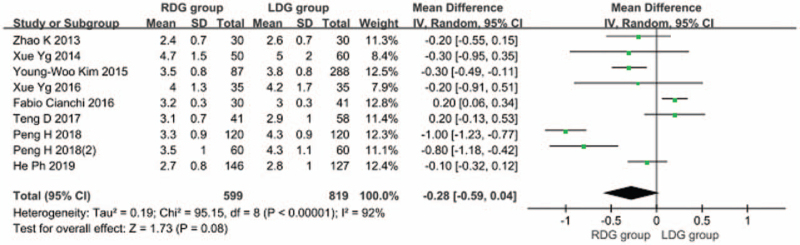
Comparison of the first postoperative anal exhaust time.

Eight articles^[7,9,10,12–14,17,18]^ reported on the first postoperative anal exhaust time, and significant heterogeneity was found between 2 studies (*I*^2^ = 92%; *P* < .1); therefore, a random-effects model was used. The findings suggested that the first postoperative anal exhaust time in the RDG group was not significantly different from that in the LDG group [WMD, –0.276 (95%CI, –0.590 to 0.037); *P* = .08] (Fig. 8).

#### 3.1.8 Length of proximal resection margin

**Figure 9:**
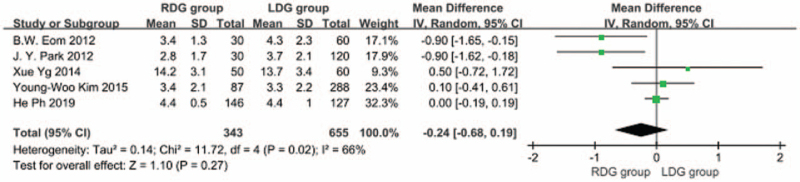
Comparison of the length of proximal resection margin.

Five articles^[5,6,9,10,18]^ reported on the length of proximal resection margin, and there was heterogeneity between 2 studies (*I*^2^ = 66%; *P* < .1); therefore, a random effects model was used. The results indicated that there was no difference in the length of the proximal resection margin between the RDG and the LDG groups [WMD, –0.245 (95%CI, –0.680 to 0.191); *P* = .27] (Fig. 9).

#### 3.1.9 Total postoperative complication rate

**Figure 10:**
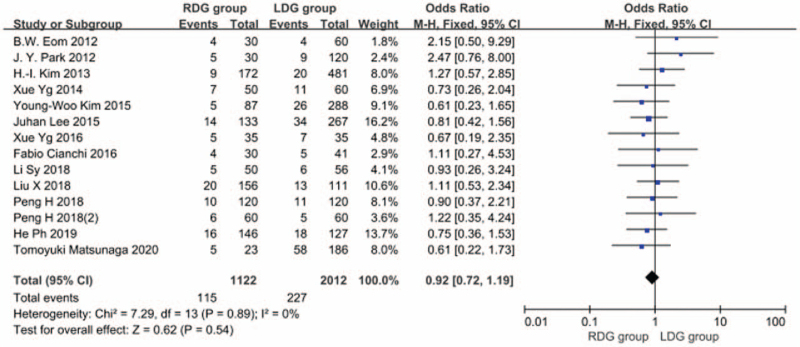
Comparison of incidence of postoperative complications.

**Figure 11:**
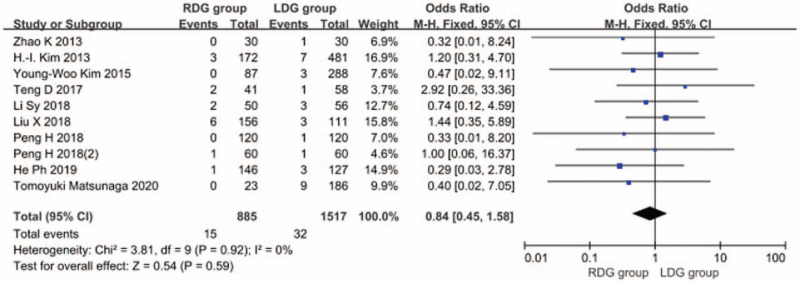
Comparison of incidence of postoperative anastomotic fistula.

**Figure 12:**
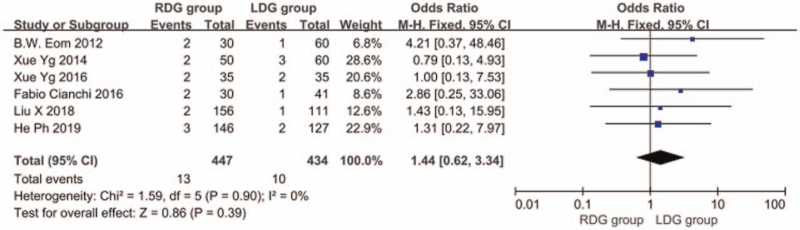
Comparison of incidence of postoperative gastrointestinal emptying disorder.

**Figure 13:**
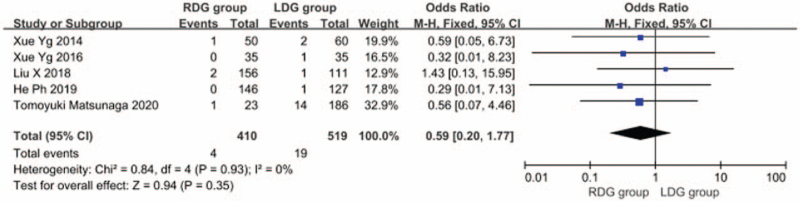
Comparison of incidence of postoperative pancreatic fistula.

Thirteen articles^[5,6,8–13,15–19]^ reported the total postoperative complication rate, and the heterogeneity between studies was significant (*I*^2^ = 0.0%; *P* > .1), and a fixed-effects model was used. The results showed that the incidence of postoperative complications in the RDG group was not significantly different from that in the LDG group (OR = 0.923; 95%CI, 0.716–1.189; *P* = .54) (Fig. 10). Among the 13 articles, 9 articles^[7,8,10,14–19]^ reported the incidence of postoperative anastomotic leakage and there was significant heterogeneity between studies (*I*^2^ = 0.0%; *P* > .1); therefore, a fixed-effects model was used. The results showed that the incidence of postoperative anastomosis in the RDG group did not differ significantly from that in the LDG group (OR = 0.841, 95%CI, 0.447–1.581, *P* = .59) (Fig. 11). There were 6 articles^[5,9,12,13,16,18]^ reporting the incidence of postoperative gastric emptying disorder, and heterogeneity between studies was significant (*I*^2^ = 0.0%; *P* > .1); therefore, a fixed-effects model was used. The results indicated that the incidence of postoperative gastric emptying disorder in the RDG group was not different from that in the LDG group (OR = 1.444, 95%CI, 0.624–3.338, *P* = .39) (Fig. 12). There were 5 articles^[9,12,16,18,19]^ reporting the incidence of postoperative pancreatic fistula, and there was significant heterogeneity between studies (*I*^2^ = 0.0%; *P* > .1); therefore, a fixed-effects model was used. The results showed that there was no difference in the incidence of postoperative pancreatic fistula between the RDG group and the LDG group (OR = 0.590, 95%CI, 0.197–1.771, *P* = .35) (Fig. 13).

#### 3.1.10 Recurrence rate

**Figure 14:**
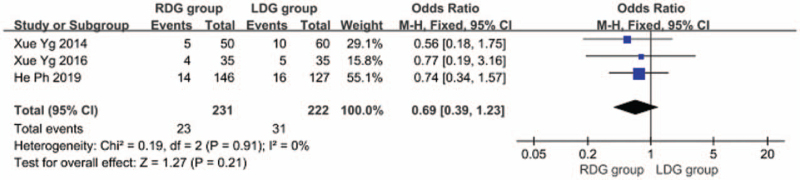
Comparison of postoperative recurrence rate.

Three articles^[9,12,18]^ reported the recurrence rate of gastric cancer in patients and the heterogeneity between studies was significant (*I*^2^ = 0.0%; *P* > .1); therefore, we used a fixed-effects model. The results showed that the recurrence rate of the RDG group did not differ significantly from the LDG group (OR = 0.689, 95%CI, 0.387–1.226, *P* = .21) (Fig. 14).

#### 3.1.11 Mortality

**Figure 15:**
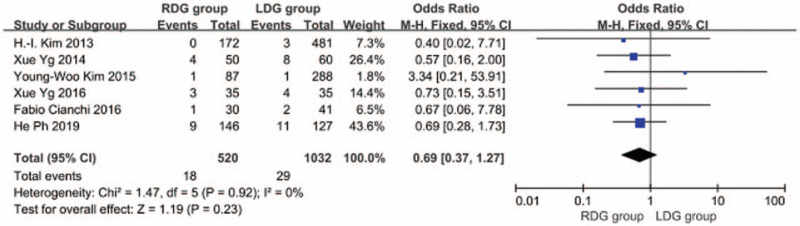
Comparison of mortality rate.

In 6 articles,^[8–10,12,13,18]^ patient quality of life was followed up for a long period of time and the mortality of patients was reported. The heterogeneity between studies was significant (*I*^2^ = 0.0%; *P* > .1), and a fixed-effects model was used. The results suggested that no significant difference was found in the mortality between the RDG group and the LDG group [OR, 0.689; 95%CI, 0.374–1.270; *P* = .23] (Fig. 15).

### 3.2 Sensitivity analysis

**Figure 16:**
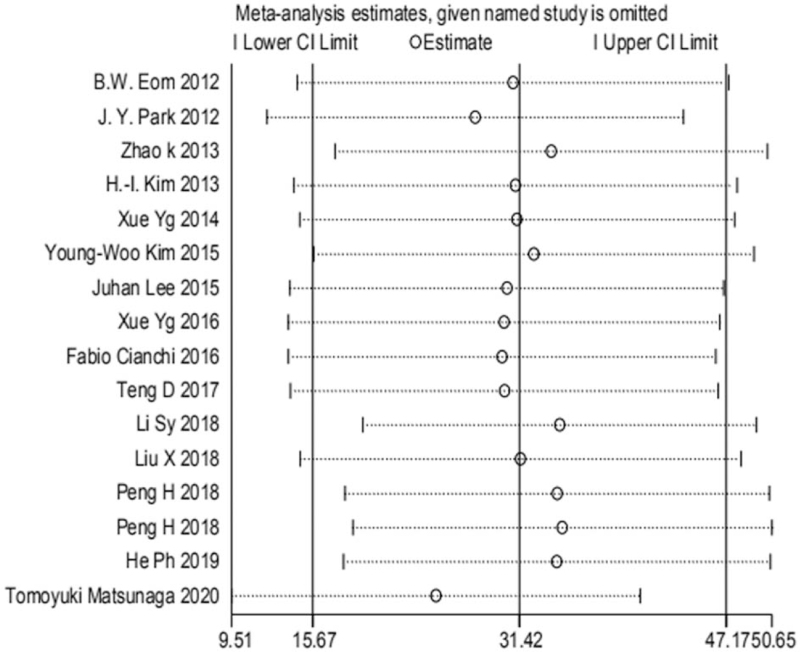
Sensitivity analysis.

The one-by-one exclusion method was used to analyze the sensitivity of all outcome indicators, and no sensitivity study was shown, indicating that the results of this analysis were stable (Fig. 16).

### 3.3 Publishment bias analysis

**Figure 17:**
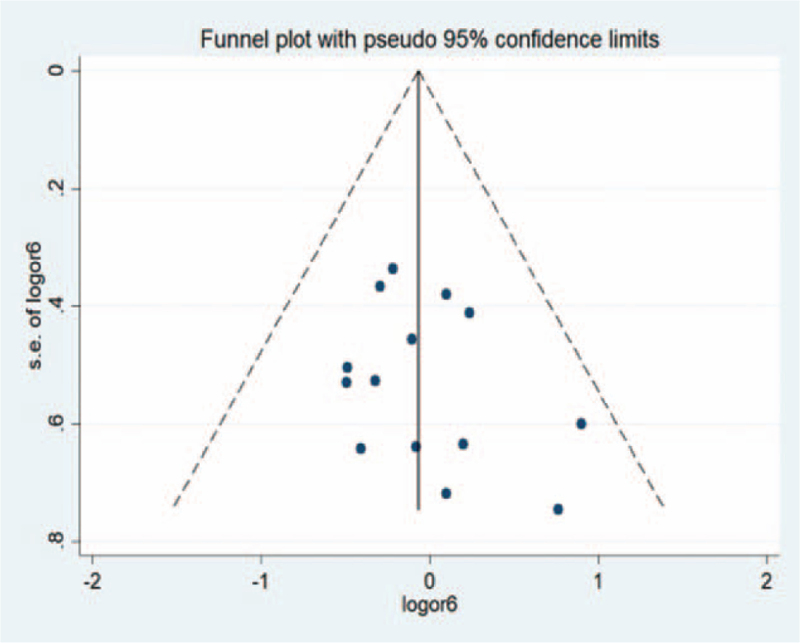
Publishment bias analysis.

The Egger test was used to analyze all the outcome indicators included in the study, and the results suggested that there was no significant publication bias in the outcome indicators of each study (Fig. 17).

## 4 Discussion

A large number of clinical studies have confirmed LDG to be a safe and radical treatment option, and the indications for surgery for early gastric cancer have been gradually expanded to include advanced gastric cancer. As a further extension of laparoscopic technique, Da Vinci robotic surgical technique has numerous advantages over traditional laparoscopic technique. Additionally, its safety and efficacy in gastric cancer surgery have also been verified by multiple studies. However, efficacy of robotic-assisted surgery, as compared with laparoscopic surgery, has not been established in high-quality studies. This study aims to provide a basis for the choice of clinical treatment via meta-analysis.

Duration of operation is one of the important indicators to measure the quality and accessibility of surgery in clinical research. The results of this meta-analysis showed that the duration of operation was significantly longer in the RDG group than in the LDG group. This might be associated with the extra time required to assemble the manipulator arms and debug the equipment, and the different learning stage the surgeon is in. Currently, in laparoscopic surgery, lymph node dissection and gastrointestinal reconstruction are time-consuming. Since all included studies in this meta-analysis focus on distal gastrectomy, the time spent on laparoscopic-assisted radical gastrectomy was shorter. In addition to the above steps that were time-consuming, the Da Vinci robotic surgical system requires an additional process of instrument assembly before RDG, and studies^[20]^ have reported that the average duration of this procedure is (62.9 ± 24.6) minutes, thereby significantly prolonging RDG. In addition, insufficient experience of surgeons in using robotic surgical systems also leads to the prolonged operation. Kim et al^[8]^ believe that the surgeon will be proficient in RG after the learning curve is overcome, thus shortening the duration of RG. Huang et al^[21]^ compared surgeons in the middle and later stages of the learning curve between RG and LG, and found that the duration of RG was shorter than that of LG. Therefore, after the learning curve is overcome, duration of RG may be significantly shortened.

In addition to the duration of operation, intraoperative blood loss is the major concerns of surgeons as one of the quality indicators. The results of this meta-analysis showed that the intraoperative blood loss of RG was significantly less than that of LG. In the included studies, the mean blood loss volume was 83.08 mL in RDG group, as compared with 111.97 mL in LDG group. This is because the robotic surgical system has inherent advantages in surgical procedure compared to laparoscopy. RDG provides surgeons with the benefit of three-dimensional operative field, which was magnified by 10 to 15 times, and helps surgeons to observe the relationship between blood vessels and surrounding tissues more directly and clearly and recognize the tissue structure. In addition, the “hands” of the robotic surgical system – the manipulator arm will help avoid unintentional tremor of human hands, which improves the stability and accuracy of the operation, thereby ensuring safety of dissection and ligation of gastric blood vessels. And studies have shown that robotic surgery can better dissect blood vessels in narrow surgical areas and reduce bleeding.^[22]^ In addition, robotic surgery will make lymph node dissection around the stomach safer and more effective. It is well-known that the focus of radical gastrectomy is whether the lymph nodes within the relevant range are thoroughly cleaned. Extensive dissection of a sufficient number of lymph nodes can not only improve the accuracy of the patient's tumor node metastasis staging, but also reduce the risk of recurrence and metastasis of patients,^[23]^ therefore, the number of resected lymph nodes is one of the important indicators of the efficacy of radical gastrectomy. This meta-analysis showed that the number of resected lymph nodes during RDG was much greater than that during LDG (25.3–41.2 vs 21.7–39.9). Therefore, RDG is superior to LDG with respect to the mean number of resected lymph nodes, and RDG can better reduce the risk of potential recurrence caused by lymph node metastasis.

Early resumption of food intake after gastrectomy for gastric cancer can help patients recover rapidly. The results of this meta-analysis showed that the time to first food intake after RDG was shorter than that after LDG. This may be partially explained by the fact that during LDG, cooperation of 2 surgeons are generally needed. Therefore, excessive intestinal canal traction may be caused due to the different traction force of the 2 surgeons in the process of tissue dissection, resulting in aggravation of intestinal stress. Blood vessels are easily damaged in this process, causing bleeding and intestinal paralysis, thereby prolonging the recovery time of intestinal peristalsis after surgery. In RDG, 4 simulated wrist instruments are available that can rotate in multiple angles in the body, which achieve the same effect of tissue traction during the operation, avoiding excessive traction of the relevant tissues, reducing the blood loss volume, and shortening the duration of intestinal paralysis. This is conducive to the recovery of intestinal peristalsis function after surgery, thereby shortening the time for the first food intake after RDG. In addition, RDG also shortens the postoperative hospital stay. The results of this meta-analysis showed that there was no significant difference in the first anal exhaust time in RDG and LDG groups, but the postoperative hospital stay in the RDG group was shorter than that in the LDG group. This indicates that given no significant difference found in the first anal exhaust time between the 2 groups, since the gastrointestinal stress of patients undergoing RDG is mild, they can consume food orally after early anal exhaust, thus speeding up the recovery of the patients after RDG and shortening the postoperative hospital stay.

In addition to the above indicators, the results of this meta-analysis indicated that the length of proximal resection margin of the RDG group was not different from that of the LDG group, but the length of distal resection margin of the RDG group was greater than that of the LDG group. The mean length of the RDG group and the LDG group was 5.3 cm and 4.6 cm respectively, which was contrary to the results of previous meta-analyses.^[24]^ According to the latest Japanese guidelines,^[25]^ the safe distance from resection margin to the lesion for invasive gastric cancer is over 3 cm, which shows that RDG is non-inferior or even superior to LDG in the radical resection of primary tumors.

In order to further analyze whether there was a disparity in the short-term prognosis of patients after RDG and LDG, in addition to the total postoperative complications, this meta-analysis also included the incidence of anastomotic leakage, postoperative gastrointestinal tract emptying disorder and pancreatic fistula, which showed no significant difference. In order to compare the long-term prognostic effects of the 2 procedures, this meta-analysis included postoperative recurrence rate and mortality. The longest follow-up time for postoperative recurrence rate was 40 months. There was no significant difference between the 2 surgical options, indicating that RDG was comparable to LDG in terms of safety and survival.

This meta-analysis has several advantages: New literature was included; All patients selected had undergone distal radical gastrectomy; The recurrence rate and mortality rate of patients after radical gastrectomy were systematically analyzed. There is no publication bias in the outcome indicators, and the conclusion is stable. The meta-analysis also has several limitations: All included articles are retrospective and high-quality randomized controlled studies are lacking; Some indicators are not included in the articles, which may reduce the credibility of the conclusion; The included articles showed incomplete data, and the positive lymph node dissection rate and the 5-year survival rate after surgery were not compared.

## 5 Conclusion

RDG is a safe and feasible procedure for distal gastrectomy, which is non-inferior or even superior to conventional laparoscopy. In view of the certain limitations of this study, a systematic analysis of multiple high-quality, multicenter clinical randomized controlled studies is needed to confirm this conclusion. With the development of medicine, RDG will be a promising approach for gastric cancer.

## Author contributions

Conceived and designed the study: Yu Liu and Zibo Zhang. Selected the studies and collected the data: Zibo Zhang, Yong Li, Qun Zhao, Xuefeng Zhao and Liqiao Fan. Analyzed the data: Zibo Zhang, Xiaolin Zhang, Zhidong Zhang, Dong Wang and Bibo Tan. Drafted the paper: Zibo Zhang. Revised the draft paper: Yu Liu and Zibo Zhang.

**Conceptualization:** Zibo Zhang, Yu Liu.

**Data curation:** Zibo Zhang, Yong Li, Qun Zhao, Liqiao Fan.

**Formal analysis:** Zibo Zhang, Xiaolin Zhang, Zhidong Zhang, Dong Wang, Xuefeng Zhao, Bibo Tan.

**Writing – original draft:** Zibo Zhang.

**Writing – review & editing:** Yu Liu.
